# Development and Evaluation of a Community Health Program to Promote Physical Activity Among Vulnerable Community-Dwelling Older Adults: Protocol for a Prospective Cohort Study

**DOI:** 10.2196/51462

**Published:** 2024-02-20

**Authors:** Tina Auerswald, Katharina Zwingmann, Torsten Schlesinger, Katrin Müller

**Affiliations:** 1 Institute of Human Movement Science and Health Chemnitz University of Technology Chemnitz Germany

**Keywords:** acceptance, ageing, aging, cognitive, cohort, communal health promotion, community based, community dwelling, community-dwelling older adults, elder, elderly, exercise, feasibility, geriatric, gerontology, observational, older adult, older people, older person, physical activity, psychosocial health, psychosocial, vulnerable

## Abstract

**Background:**

Vulnerable older adults have a high risk of morbidity and mortality. Regular physical activity (PA) can have a positive effect on the health and health-related behavior of this specific target group. However, evidence of the impact and feasibility of community-based PA promotion interventions for vulnerable older adults is still limited.

**Objective:**

The BeTaSen (Bewegungs-Tandems in den Lebenswelten Chemnitzer Seniorinnen und Senioren: ein Beitrag zur kommunalen Gesundheitsförderung) study aims to evaluate the (1) impact as well as the (2) feasibility, acceptance, and usefulness of a 12-month low-threshold PA intervention program for community-dwelling vulnerable older adults.

**Methods:**

For our population-based prospective observational cohort study, a total of 120 vulnerable older adults (aged 75 years or older) in the area of Chemnitz (Germany) will be recruited to participate in (1) weekly neighborhood-based low-threshold PA meetings with trained mentors (activity tandems) and (2) monthly exercise meetings led by trained exercise instructors. Within the intervention, participants will be encouraged to perform the PA independently. Participants will complete assessments, which will include questionnaires as well as objective measurements of their physical, cognitive, and psychosocial health at 3 different time points (baseline, 6 months after the start, and 6 months after the end of the intervention). Additionally, a process evaluation will be performed, including questionnaires and qualitative interviews, involving the participants, mentors, and municipal project partner representatives.

**Results:**

The BeTaSen project process began in October 2021, with the start of data collection and intervention in August 2022 in the first neighborhoods of the city of Chemnitz. A total of 86 participants were recruited at the time of submission of the manuscript. Longitudinal results are expected by 2025.

**Conclusions:**

This study’s results will provide insights on (1) the PA behavior of vulnerable older adults as well as the impact of PA interventions on health-related outcomes such as cognitive, physical, and psychosocial health, and (2) the feasible and useful components of community-based PA interventions. Thus, this pilot study contributes to future recommendations and provides a basis for further research, such as the development of feasible and sustainable target group–specific interventions in community settings.

**International Registered Report Identifier (IRRID):**

DERR1-10.2196/51462

## Introduction

### Overview

The share of older adults in many European countries’ total population is increasing [1]. Aging is associated with a number of physical and health-related changes, including a decline in physical and cognitive performance and an increase in neurodegenerative as well as cardiovascular diseases [2]. Furthermore, demographic developments and age-related increases in individual disease risk pose significant (health-related) economic and social challenges for aging societies. This manifests itself in increased hospitalizations [3] and financial burdens on both the health care system and private households due to rising health care costs [4]. Thus, healthy aging, for example, years of life spent in good health and the maintenance of well-being in old age, has enhanced attention in social and health systems [2]; investments in and programs to promote health are increasing in importance. According to current research, vulnerability in older adults (eg, poverty, loneliness, and a low level of education) can adversely affect the health behavior, morbidity, and mortality of older adults [5-9]. German adults with an income of less than 60% of the national average, for example, have a significantly reduced life expectancy compared to those with above-average incomes [8]. Consequently, it is crucial to focus, especially on these target groups, on health promotion programs.

Among other health-promoting interventions, recent studies confirm that regular physical activity (PA), for example, daily walks, can improve individual and biopsychosocial health among healthy as well as vulnerable older adults [10-16]. Moreover, regular PA can also have a positive impact on social connectedness [13], which in turn can improve older adults’ cognitive and mental health and seems to be a relevant factor in successful aging [14]. Accordingly, regular PA can be considered a beneficial investment in individual health capital [15] and in healthy aging in general [11]. Furthermore, previous studies and systematic reviews have shown that PA interventions in community settings can improve older adults’ physical function and level of PA [16-20], even in those who are vulnerable [16,20]. For example, Giné-Garriga et al [16] find that physical exercise interventions can improve physical function in frail older adults. Other studies conclude that such programs can have positive effects on older adults’ cognitive and mental health [21,22]. Recently, studies investigated the impact of community-based PA interventions on older adults’ cognitive, psychosocial, and physical health and on their PA behavior in German communities [23,24]. However, many studies involved mostly healthy older adults who did not belong to a vulnerable group, and current research does not address long-term implications or specific group differences.

Thus, more evidence is needed to confirm these positive impacts, especially on vulnerable older adults, and to implement subsequent health promotion programs. Furthermore, despite the well-known positive effects of regular PA, only a small fraction of older adults in Germany are sufficiently physically active in line with the recommendations of the World Health Organization (WHO) [25]. The WHO and the American College of Sports Medicine recommend adults aged 65 years or older perform moderate to vigorous endurance training for a minimum of 150 minutes per week (in bouts of at least 10 minutes each) [26], in addition to flexibility, strength, and balance training twice per week [25]. Among adults aged 65 years or older, 42% meet the recommendations for endurance training, while only 29% meet those for strength training [25]. In a German health monitoring survey, 36% of older adults aged between 60 and 69 years and nearly 45% of older adults aged between 70 and 79 years reported that they do not engage in any regular PA or exercise [27]. Several studies find that vulnerability, for example, a low level of education, physical restrictions, and poor health status, is negatively associated with older adults’ PA level (eg, meeting the WHO’s recommended level of PA) [25]. This specific group is thus exposed to severe health risks and potentially early care dependency [5-8]. Due to the high share of older adults who are physically inactive, feasible PA promotion programs targeted specifically at previously physically inactive or vulnerable older adults are especially relevant. To determine the feasibility and sustainability of PA programs targeted specifically at vulnerable older adults, further research on contributing factors and barriers is needed. The literature identifies several internal and external barriers for older adults’ participation in PA programs: lack of information about PA offers, physical restrictions, lack of social support to begin with PA, lack of language skills and local accessibility, as well as financial costs [28]. Vulnerable older adults (eg, poverty, loneliness, and a low level of education) perceive high barriers to participation in health-related promotion programs, for example, PA programs [29]. In a systemic review, Franco et al [30] identified six major themes that may influence older adults’ participation in PA: (1) social influences, (2) physical restrictions, (3) competing priorities, (4) access difficulties, (5) lack of awareness of the personal benefits of PA, and (6) motivation and beliefs [30]. Furthermore, current research recommends providing PA interventions in community settings for vulnerable older adults that are specifically tailored to the target group and embedded in their immediate social-spatial context (districts and neighborhoods) [31-33]. Moreover, previous studies have shown that personal interventions are particularly effective in promoting PA [34] and that motivators such as social and environmental support, convenience of location, and enjoyment of the activity seem to be relevant factors in interventions targeted at community-dwelling older adults [35]. These influencing factors should be considered when developing exercise interventions to promote PA and its associated positive health benefits among vulnerable community-dwelling older adults.

Some studies conclude that community-based PA programs for older adults are feasible, even over a longer period [36-38]. A systematic review carried out by Farrance et al [39], for example, finds long-term adherence rates of nearly 70% for community-based group exercise programs. Nevertheless, there is limited evidence about the long-term adherence measures included in these programs as well as participants’ views [39]. Shvedko et al [40] assert that a PA intervention for community-dwelling older adults at risk of loneliness is accepted by older adults and is feasible. Crombie et al [41] also show that a community-based PA intervention is feasible for sedentary older adults. However, recent evidence about the feasibility and acceptance of community-based PA interventions and of the different components of such interventions, especially among vulnerable older adults, is limited.

In summary, evidence of the impact and feasibility of PA interventions on the health and health-related behavior of vulnerable community-dwelling older adults remains sparse. Therefore, research should focus on the health-related benefits of intervention programs that promote PA among vulnerable older adults as well as on how intervention programs tackle (internal and external) barriers that discourage the participation of vulnerable older adults in PA programs.

For this reason, the aim of this study is to investigate the impact and feasibility of a 12-month low-threshold targeted PA intervention program on community-dwelling vulnerable older adults. This study protocol describes the study’s objectives, the design of the intervention, method, data collection, and considerations for analyzing the data from the PA intervention program of the study BeTaSen (Bewegungs-Tandems in den Lebenswelten Chemnitzer Seniorinnen und Senioren: ein Beitrag zur kommunalen Gesundheitsförderung).

### Research Objectives and Research Questions

The main goal of the BeTaSen study is to initiate, sustainably maintain, and promote an active and healthy lifestyle for vulnerable older adults who reside in Chemnitz.

The objectives of the BeTaSen study are described in subsequent paragraphs.

Evaluation of the impact of a 12-month low-threshold PA intervention program on health-related outcomes of vulnerable community-dwelling older adults. In this context, the main research questions are as follows: (1) What are the long-term outcomes for older adults after completing a 12-month, target group-specific community-based PA program? Older adults’ PA behavior as well as their physical, cognitive, and psychosocial health are of particular interest in this regard. (2) Are any group differences evident in the long-term outcomes of a 12-month target group-specific community-based PA program among older adults (eg, gender, previous disease, and vulnerability)?

Analyzing the feasibility, acceptance, and usefulness of aspects of a community-based PA intervention for vulnerable community-dwelling older adults. In this context, the main research questions are as follows: (1) Which PA-promoting exercises and activities are considered useful by older adults? (2) Is a target group-specific 12-month PA program feasible for vulnerable community-dwelling older adults and the community setting? and (3) What factors promote or inhibit the successful implementation of a target group-specific PA program for vulnerable community-dwelling older adults?

## Methods

### Participants and Procedure

The pilot study is being implemented in the German city of Chemnitz. Chemnitz is the third-largest city in the federal state of Saxony in the southeastern part of Germany, with a population of 250,398 (in 2023) [42]. In 2022, nearly 30% of the population of Chemnitz was aged 65 years or older; 15% were aged 75 years or older [43]. In 2020, Chemnitz was the city with the highest median age in Europe (52 years) [44]. Forecast calculations expect that by 2030, the Chemnitz region will have the highest share of people aged 65 years or older (nearly 38%) in Europe [1]. According to population forecasts, the share of Chemnitz residents aged 80 years or older will also rise from 8% in 2018 to nearly 11% in 2030 [45]. Due to these demographic developments, there is an urgent need for target group-specific and low-threshold community health promotion programs (eg, PA interventions) to support the healthy aging of vulnerable older adults who live in the city of Chemnitz. This city was furthermore chosen due to its immediate proximity to the Technical University of Chemnitz, which is conducting the study, and its collaboration with the city of Chemnitz’s health department, as well as support from various local partners (eg, citizen platforms).

The aim during the study period is to cover at least 4 out of 39 districts in Chemnitz, representing the city’s average demographic age (>50 years) and lower socioeconomic districts (compared to the city of Chemnitz’s average) [46,47]. The recruitment of participants is planned primarily due to collaboration with local partners. Announcements will be conducted in different communal facilities for older adults (eg, citizen online platforms, assisted living facilities, and citizens’ meeting points) through flyers, at information events, in institutional newspapers, and in the personal conversations of the research team with targeted older adults as well as with staff of different social institutions. The study will be announced in various local and district newspapers, and flyers will be distributed in shops, for example, in bakeries and pharmacies. Additionally, older adults who had participated in previous studies and had consented to being informed about future studies will be contacted.

Eligibility to participate in the study will be determined in personal interviews by qualified staff based on the inclusion and exclusion criteria described below.

### Inclusion and Exclusion Criteria

The study primarily targets older adults aged 75 years or older who can be considered vulnerable (eg, poverty, loneliness, physical restrictions, and a low level of education) and who reside in the city of Chemnitz.

Residents are eligible to participate in the study if they (1) have at least basic knowledge of the German language, (2) can independently travel to the assessment and intervention locations, which will be meeting points in close proximity to their home, and (3) provide informed consent to participate in the study.

Participants will be excluded from the study if they have a medical condition preventing them from engaging in regular PA or if they had a severe adverse health event within the last 6 months (eg, myocardial infarction, stroke, fracture, surgery, or pneumonia) to ensure their safety during the PA sessions. Additionally, participants will be excluded if they participate in other clinical trials as well as in regular PA programs at least twice a week (eg, regular gym visits or rehabilitation exercise programs) to address older adults who are less physically active and to analyze solely the impact of the intervention.

### Study Design

**Figure 1:**
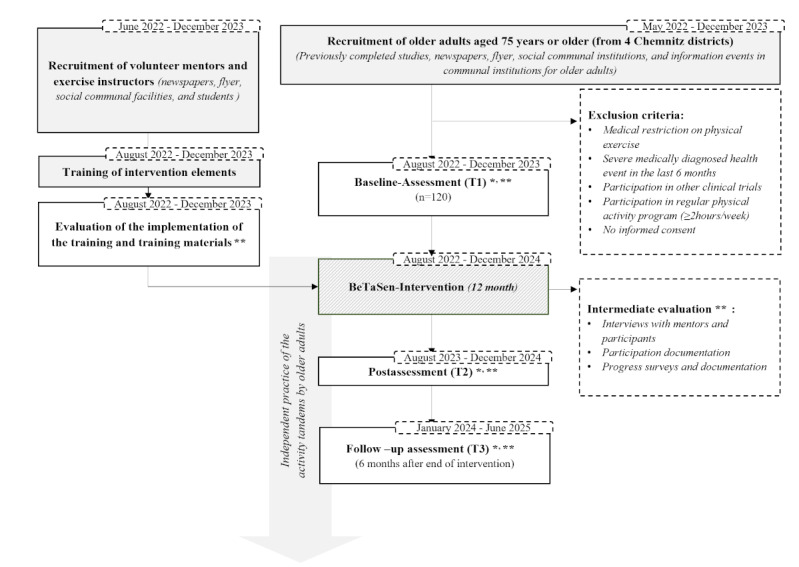
Study flow of the population-based prospective observational cohort study BeTaSen (Bewegungs-Tandems in den Lebenswelten Chemnitzer Seniorinnen und Senioren: ein Beitrag zur kommunalen Gesundheitsförderung) with a study population of older adults (aged 75 years or older) living in Chemnitz, Germany. *Outcome evalution. **Process evaluation.

The pilot study is designed as a population-based prospective observational cohort study and is being carried out in 4 different districts in the city of Chemnitz. The first screening for study eligibility will be assessed through a standardized telephone interview with, for example, questions about older adults’ age, health conditions, and regular weekly PA exercises. Afterwards, participants will complete an assessment conducted by the research team in their direct neighborhood (eg, in the facilities of community centers), which includes questionnaires and objective measurements of physical and cognitive function and of psychosocial health. In addition to these measurements, participants will wear a pedometer for 7 consecutive days to assess their PA behavior. Measurements will take place at 3 different time points over an 18-month period, namely at the beginning (T1), at the end (T2), and 6 months after the end of the intervention (T3) (Figure 1).

The project was launched in October 2021 and will be completed in September 2025. The first participants were recruited in August 2022.

### Design and Organization of a Physical Activity–Promoting Intervention

**Figure 2:**
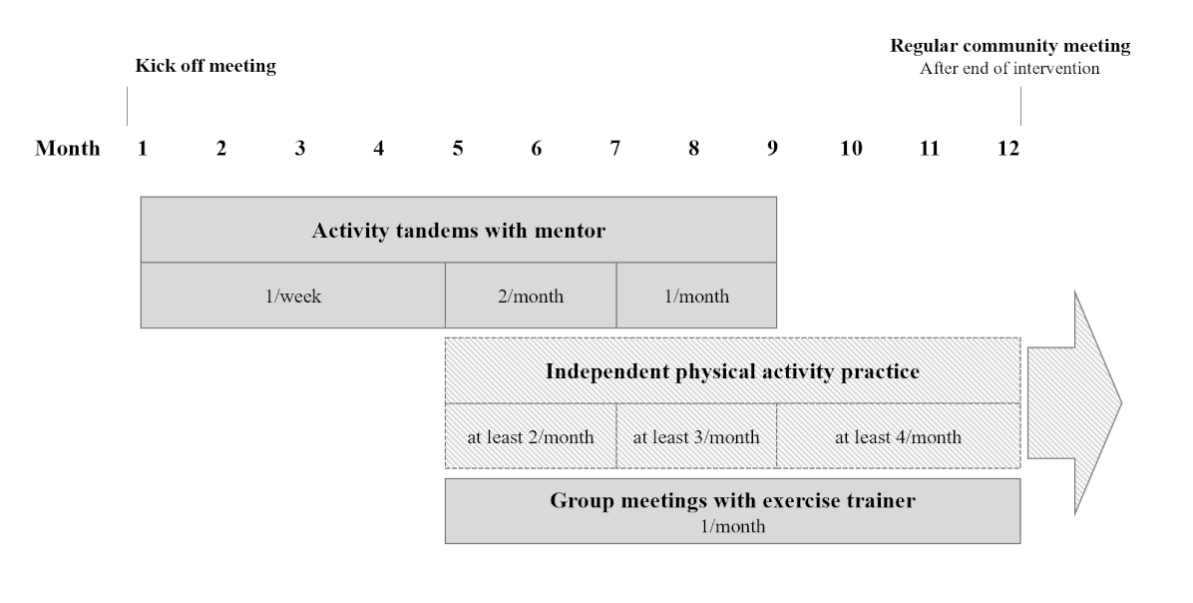
Procedure of the population-based prospective observational cohort study BeTaSen's intervention over a period of 12 months; an example is shown for 1 district (study population: older adults aged 75 years or older living in Chemnitz, Germany). BeTaSen: Bewegungs-Tandems in den Lebenswelten Chemnitzer Seniorinnen und Senioren: ein Beitrag zur kommunalen Gesundheitsförderung).

All participants will participate in the target group-specific community-based PA intervention. The pilot study’s intervention period is 12 months per participating neighborhood. The participants will be encouraged to continue to engage in PA independently (Figure 2).

The intervention’s main component will comprise regular PA meetings organized in so-called “activity tandems,” which take place once a week for 30-45 minutes. One activity tandem will include 1 trained voluntary mentor and 3-4 older adults living in the same neighborhood. These activity tandems will primarily engage in low-threshold outdoor PA and exercise together in their neighborhood. For this, easily accessible locations such as urban lawns, walking paths, parks, and fitness trails will be used. In case of bad weather, the PA meetings will be organized in the meeting places of collaborating partners, for example, community centers. Due to the small group size, the content of each tandem lesson can be flexible and tailored to the individual participant’s needs. For example, the exercises can be performed at different levels of intensity, or alternative exercises can be selected from a large PA-exercise pool. Additionally, monthly group meetings (eg, 3-4 activity tandems with a total of 12-16 older adults) will be organized in each neighborhood district in the intervention months 5-12 (Figure 2), which will be delivered by trained exercise instructors. In these group meetings, in addition to PA exercises, the following contents will be covered: knowledge about the positive effects of PA, goal setting and planning, barrier management, and motivational aspects, as well as the sustainable implementation of regular PA in everyday life. Measures like small group sizes, qualified mentors, support material (eg, first aid kits, pulse oximeters, Borg scales), and the accessible, familiar environment of the activity sessions ensure the safety and well-being of participating older adults.

In total, around 6-8 activity tandems will be created in each of the 4 local districts (approximately 24-32 older adults per district). Thus, the goal is to reach a total of approximately 120 older adults residing in the city of Chemnitz during the project period.

Voluntary mentors (eg, volunteers from Chemnitz, students, and community center staff) will be recruited through various public channels (eg, newspapers, flyers, and mailing lists) in cooperation with municipal partners. The trained mentors should guide the activity tandem groups (eg, demonstrate PA exercises with the help of the manual and walk paths), ensure safety and well-being (eg, through individualized feedback), and encourage the older adults to do independent regular PA exercises. The mentors will also be responsible for communication and the planning of the activity tandem meetings. Researchers on the project team will instruct mentors in a workshop about how to conduct PA meetings, how to appropriately deal with the target group, and how to perform basic first aid procedures. In addition, mentors will be equipped with support materials, such as a manual that includes a range of various low-threshold PA exercises, an overview of district walkways, and additional exercise materials (eg, resistance bands and everyday objects).

The mentors will supervise and support the activity tandems once a week initially at the start of the intervention (intervention months 1-4), later once every 2 weeks (intervention months 5-6), and then once a month (intervention months 7-9). The older adults will thus be gradually encouraged to independently engage in weekly PA activity meetings, as from intervention month 10 onward, they will be prepared for basically independent implementation. Furthermore, older adults will be encouraged to actively participate in the activity tandems, for example, by choosing walkways or suggesting individual exercises. During the intervention period, participants will be given exercise material (eg, brochures) to continue regular PA in their activity tandem and on their own. To ensure that older adults do not overexert themselves as well as to enable them to get a better sense of their exercise limits, their oxygen saturation and heart rate (using a pulse oximeter; Orbisana Healthcare GmbH) as well as a subjective exertion rate (based on the Borg scale; [48]) will be measured at the beginning, middle (during physical exertion), and end of each activity tandem meeting. Based on these measures, participants will receive direct feedback, and the PA exercise intensity can be adjusted individually.

In line with previous literature and findings, the vulnerable target group’s potential barriers should be carefully considered when developing the pilot study. To ensure low-threshold accessibility, the activity tandems and monthly group meetings will take place in the older adults’ neighborhoods. District plans will be drawn up to identify sitting areas and public toilets, for example, and to pinpoint accessible walkway plans. The activity tandem’s PA content will be designed to reflect daily life activities in a very playful, individualized, ability-oriented, and motivational way. Each unit can be freely designed by the mentors and older adults based on the PA, exercise materials, and recommendations provided. Each activity tandem unit should begin with a warm-up, and the components of strength, endurance, coordination, stretching, and relaxation should be addressed regularly in different sessions. In addition, the mentor should include knowledge transfer in each unit, for example, about the positive effects of PA. Moreover, participation in the PA intervention does not entail any financial costs (eg, materials and participation fees). To promote sustainable participation in the neighborhood meetings and to ensure that the PA meetings are tailored to the vulnerable target group, the activity tandems are purposely organized for a smaller number of older adults. The older adults and mentors of the activity tandems can coordinate the time and location of their weekly meetings flexibly on their own.

### Measures

All participants will complete an assessment at baseline (T1), at the end of the 12-month intervention (T2), and 6 months after the end of the intervention (T3). Before each assessment, participants will be asked to fill out a self-administered questionnaire and, for the detection of objective PA behavior, will be given a pedometer to wear for 7 consecutive days. The questionnaire will assess the older adults’ psychosocial and physical health and evaluate motivational aspects related to PA in a community setting. Sociodemographic information will be assessed within the questionnaire at T1 only, comprising target group-specific items from the German Health Interview and Examination Survey for Adults [49].

Further measurements will take place in communal facilities in the older adults’ neighborhood and will be carried out by qualified staff members. Each assessment will start with the measurement of anthropometric data (height and weight) as well as the registration of current medication intake and existing diagnosed diseases via interview. Using the EQ-5D-3L questionnaire, the participants’ subjectively perceived health status in terms of mobility, self-care, regular daily activities, pain, discomfort, anxiety, and depression will then be determined by interview (3 response options: no problems, some problems, and serious problems) [50]. Additionally, their general self-rated health status will be recorded using the vertical visual analog scale EQ-VAS (1-100) [50].

An additional process evaluation will be performed during the intervention period using questionnaires for participants (after a period of 4 weeks) and mentors (after the workshop, 3 months after the intervention). Furthermore, qualitative interviews and informal notes, reports, and documentation of the PA groups will also be obtained from the activity tandems as well as from group meetings (Table 1 for a summary of outcomes).

**Table 1:** Outcome measurements of the population-based prospective observational cohort study BeTaSen (Bewegungs-Tandems in den Lebenswelten Chemnitzer Seniorinnen und Senioren: ein Beitrag zur kommunalen Gesundheitsförderung). T1 is baseline, T2 is the end of the intervention period (12 months), and T3 is 6 months after the end of the intervention (study population: older adults aged 75 years or older living in Chemnitz, Germany).

Assessment		Instrument or scale	Time of measurement	
**Outcome**				T1, T2, T3
	Physical function	Short Physical Performance Battery (SPPB) Handgrip strength (handgrip dynamometer)		
	Physical activity	OMRON Walking Style One 2.1. Pedometer (7 days) PRISCUS-PAQ		
	Psychosocial health	German Depression Scale (DIA-S) Items of Tilburg Frailty Indicator (TFI) Items of social support questionnaire (F-SozU)		
	Cognitive function	Montreal Cognitive Assessment (MoCA)		
**Process**				
	Feasibility	Documentation of activity tandems and group meetings adherence rates Qualitative interviews and self-administered questionnaire with older adults, mentors, exercise trainers, and communal partners	Each activity tandem or group session Monthly during the activity tandem period and after the end of the individual intervention period (older adults); after the end of intervention in the respective neighborhood (mentors, communal partners)	
	Usefulness	Qualitative interviews and self-administered questionnaire with older adults, mentors, and exercise trainers	Monthly during the activity tandem period and after the end of the individual intervention period (older adults); after the end of intervention in the respective neighborhood (mentors, communal partners)	
	Implementation (barriers and facilitators)	Qualitative interviews and self-administered questionnaire with mentors, exercise trainers, and communal partners	Monthly during the activity tandem period and after the end of the individual intervention period (older adults); after the end of intervention in the respective neighborhood (mentors, communal partners)	

The main activity and health-related outcomes will be addressed in the following subsections.

#### PA Behavior

PA behavior will be objectively assessed using the OMRON walking style one 2.1. pedometer (OMRON Cooperation) that records a person’s steps, walked kilometers, kilocalories used, and aerobic steps per day over 7 consecutive days. Participants will be instructed to wear the pedometer on their hip during waking times as well as get information about the use and purpose of the pedometer. After the pedometer has been returned, the data will be transferred by study assistants to a Microsoft Excel (Microsoft Corporation) spreadsheet. Activity data for days with at least 500 steps will be included for further analysis, according to Meyer et al [51].

For a subjective evaluation of PA behavior, the PRISCUS-Physical Activity Questionnaire (PRISCUS-PAQ) for older adults aged 70 years or older will be assessed based on interviews during T1, T2, and T3 measurements [52]. PRISCUS-PAQ consists of 10 main items on sedentary behavior, daytime naps, cleaning, and other housework, cycling, gymnastics, group exercise, other PA, walks, and gardening. The total score of the PRISCUS-PAQ reflects the individual’s energy expenditure (metabolic equivalent) during the previous week [52].

#### Physical Performance

The digital grip dynamometer (JAMAR Smart Hand Dynamometer; Performance Health Supply Inc) will be used to assess the participants’ handgrip strength. During this test, participants sit upright in a chair without an armrest with their arm bent to 90°. A total of 3 trials are performed per hand, alternating between right and left, with a test duration of 3-5 seconds for each trial. The mean and highest values of the 3 trials for each hand will be analyzed [53]. Additionally, the dominant hand will be queried.

To prove the impact of the exercise intervention on participants physical performance, the widely used, highly reliable, and valid Short Physical Performance Battery (SPPB) will be used [54]. The SPPB includes balance (Romberg, Semi-Tandem, and Tandem), gait speed (4-meter walk), and chair rise tests. Each of these 3 SPPB components is scored from 0 (inability to carry out) to 4 (best performance). There is a potential range between 0 and 12, with higher scores indicating higher functional performance. Participants with cut-off points of less than or equal to 6 points will be classified as “poor performers.” Whereas a score of 7-9 points will define the “moderate performers,” and the “good performers” will have scores of 9 points or more [55]. In a systematic review and meta-analysis, the SPPB has been shown to be a predictive tool for all-cause mortality [56].

The 2-minute step test (TMST) [57] will be used to assess the participants’ endurance capacity. The test starts with a 2-minute rest period. Participants will then stand in front of a wall and raise their thigh to be horizontal to the floor; a mark (strip) will be placed in the position corresponding to the middle of their thigh bone, between the kneecap and frontal prominent pelvis bone. Participants will be instructed to alternately lift their knees as often as they can over a 2-minute period, such that their knees reach the marked location. After completing the TMST, the researcher will record the participants’ number of steps, or “steps,” as an outcome measure. Before the start of the test, directly after, and 30 seconds after the end of the test, the participant’s heart rate in beats per minute and measurement of oxygen saturation in percent (through a pulse oximeter) as well as a subjective rating of perceived exertion (through the Borg scale [48]) will be recorded.

#### Cognitive Function

The short screening tool Montreal Cognitive Assessment (MoCA) will be used to evaluate the participants’ cognitive functions (eg, memory, attention, and basic functions). Participants who achieve a MoCA score between 26 and 30 points will be classified as cognitively healthy; those with a MoCA score of 19-25 will be considered to have mild cognitive impairment; and participants with a MoCA score of less than 19 will be defined as severely cognitively impaired [58].

#### Psychosocial Health

Psychosocial health outcomes will be assessed using modified, validated, and reliable instruments included in a self-administered questionnaire. Thus, the psychological and social domains of the Tilburg Frailty Indicator [59], the German depression scale for older adults, namely Depression im Alter Skala [60], and modified items of the Fragebogen zur sozialen Unterstützung (social support) [61] will be implemented.

#### Feasibility and Usefulness

The intervention’s feasibility for older adults will be assessed quantitatively based on the adherence rate (number of completed activity tandems and group meetings) during the intervention period. Additionally, participants will be given their own documentation sheet to record their subjective well-being (using a 5-level smiley scale at the beginning, middle, and end of each session) and their level of satisfaction (using a 5-point Likert scale) with each activity tandem meeting. Additional self-administered questionnaires with items to subjectively rate the activity tandem’s components will be implemented after every fourth unit. Using a 3-point Likert scale, participants will rate the difficulty of the particular intervention content (eg, strength, endurance, and coordination). In addition, they will be able to use the open response section to indicate which parameters they want to improve and what they want to achieve over the next 4 weeks. Six months into, at the end, and 6 months after the end of the intervention, participants will receive another self-administered questionnaire to assess the intervention’s feasibility and sustainability and to identify the optimizing factors of the activity tandems and group meetings.

To evaluate the feasibility of volunteer mentors and the communal context, (1) mentor workshops will be anonymously evaluated using a self-administered questionnaire, and (2) mentors and community project partner representatives will be asked to fill out a self-administered questionnaire (eg, questions on perceived feasibility and sustainability).

#### Implementation

To assess the implementation of the PA intervention, (1) self-administered questionnaires and (2) qualitative interviews will be conducted with mentors, participants, and community project partner representatives.

### Data Management

Participant information will be recorded using an individual identification code. All hard copies will be stored at Chemnitz University of Technology in locked cabinets accessible only to project staff members. Electronic data will be stored on a secured, password-protected computer. The databases will not contain participant identifiers. The data linking participant identifiers and the individual identification codes will be stored separately. Data quality will be ensured through double data entry and range checks for data values. Only project staff members will have access to the final trial data set.

### Statistical Analysis

SPSS Statistics (version 29; IBM Corp) and AMOS (version 29; IBM Corp) will be used for all quantitative statistical analyses. To determine the primary outcome at baseline (T1), the study population will be descriptively characterized using the following outcomes: demographics (eg, age, gender, family status, and income), anthropometric comorbidities, PA and health-related behavior (eg, smoking status and alcohol consumption), as well as physical, functional, cognitive, and psychosocial health status. Furthermore, participants’ data at the end of the intervention period (T2) and 6 months after its end (T3) will be analyzed to evaluate the long-term impacts, feasibility, and usefulness of the PA intervention. Time effects, group differences (eg, age, gender, vulnerability, and sociodemographic conditions) in terms of physical, cognitive, and psychosocial health, interaction effects, as well as the intervention’s feasibility and sustainability, will be analyzed using different parametric or nonparametric statistical methods such as the *t* test, Mann-Whitney *U* test, ANOVA, or Kruskal-Wallis test, depending on the number and size of the group, normal distribution, and homoscedasticity of the sample. Based on current evidence [6-8,25], associations and the strength of the relationship between 2 variables (eg, PA behavior and monthly income and adherence rate and level of education), correlation and regression analyses as well as adjustments for relevant covariates (eg, age, gender, and vulnerable criterion) will be performed. Additionally, multilevel analyses are planned to estimate, for example, the within-person impacts of the intervention program (eg, the impact of PA on well-being).

Qualitative interviews and focus group discussions will be protocolized by researchers and audio recorded, which will be transcribed afterwards. Qualitative data will be analyzed using a qualitative content analysis, according to Mayring and Fenzl [62], with the aim of structured processing of the material by coding relevant interview passages deductively in a theory-driven system of categories. Frequent text material that cannot be assigned to a deductive main category will result in the creation of new (inductive) main categories or subcategories [62]. The coding scheme will subsequently be supplemented with additional categories that emerge from the text material. These newly added categories will either be treated as subcategories of already existing categories or as new main categories. The text material will be coded with the help of MAXQDA (VERBI GmbH).

For this pilot study in the municipal area, the number of participants was calculated based on the project period and the project funding type in terms of feasibility within the study period.

### Ethical Considerations

This study was approved by the Ethics Committee of the Chemnitz University of Technology, Faculty of Behavioral and Social Sciences, on May 19, 2022 (101547973). All study participants will be fully informed about the objectives and procedures of the study in German and will be requested to give written informed consent. Additionally, the informed consent contains information about the pseudonymized data collection for the study. Furthermore, the original informed consent allows the secondary analysis without additional consent.

## Results

The intervention period of the BeTaSen study started in August 2022 in the first neighborhoods of the City of Chemnitz. Due to the planned staggered launches of the different activity tandems, cross-section data of all participants at T1 (approximately 120 older adults) and initial feasibility data 6 months into the intervention (approximately 60 older adults) will be collected until December 2023. Longitudinal results are expected in the first quarter of 2025. Qualitative and quantitative data on sustainable components for PA interventions among all participating vulnerable older adults are expected in December 2024, after T2, and 6 months after the end of the intervention (T3).

## Discussion

The aim of the BeTaSen study is thus to develop, implement, and evaluate a target group-specific PA intervention for community-dwelling older adults living in Chemnitz, the city with the highest median age in Europe [44].

The intervention program was developed especially for the target group of vulnerable community-dwelling older adults on the basis of theoretical aspects [29-35] and considers potential barriers and facilitating factors for initiating and maintaining regular PA. Furthermore, the specific intervention design contains elements promoting long-term use (eg, increasing independent activity sessions over the intervention period, individual goal setting, and knowledge transfer of the effects of PA). Therefore, the intervention concept and their implementation are expected to contribute to (1) increasing or maintaining PA behavior, cognitive and physical function, as well as psychological parameters of older adults, and (2) the feasibility and sustainability of healthy aging in municipalities.

The results of the study will provide insights into (1) the PA behavior of vulnerable older adults as well as the impact of PA interventions on health-related outcomes such as cognitive, physical, and psychosocial health, and (2) feasible and useful components of community-based PA interventions. Thus, this pilot study bridges a research gap as it focuses on the PA-promotion of vulnerable community-dwelling older adults and implements a low-threshold health promotion concept that may have a sustained impact on the health and health-related behavior of this specific target group. Furthermore, the BeTaSen study will make an important contribution to the state of knowledge of average PA levels and health outcomes, as well as the impact, feasibility, and sustainability of such interventions. As the results of the study will provide insight into the demands, needs, and possible barriers of programs, it can provide a foundation for new interventions and how to deal with difficulties in their implementation.

During and at the end of the project period, interim results and findings will be summarized in information brochures and presented at municipal information events or scientific congresses. When interpreting the results, it should be considered that this research project does not have a randomized, controlled study design since the accessibility of the target group is very restricted and community-based PA programs should be accessible to vulnerable older adults over the entire study period. Nonetheless, the results of the study will provide insights into the within-person impacts of regular PA over a longer time period, and the study design comprises a low-threshold intervention for a highly relevant target population and allows “real-world” applicability. The results of the study will make an important contribution to the research field of aging and movement sciences as well as to the development of health-promoting structures, providing insights into the impact and implementation strategies of community health promotion programs for specific target groups like vulnerable older adults. In addition to publications in scientific journals, the impulses of the study will be implemented into municipal health policies and structures (eg, other neighborhoods, community centers, and health departments) after the end of the project period. Furthermore, the PA program can be distributed throughout Germany through cooperation with the Federal Centre for Health Education and thus shows a high potential for expansion.

Finally, the results will contribute to future recommendations and provide a basis for further research, such as the development of feasible and sustainable target group-specific interventions in community settings.

## Abbreviations

BeTaSen: Bewegungs-Tandems in den Lebenswelten Chemnitzer Seniorinnen und Senioren: ein Beitrag zur kommunalen Gesundheitsförderung
MoCA: Montreal Cognitive Assessment
PA: physical activity
PRISCUS-PAQ: PRISCUS-Physical Activity Questionnaire
SPPB: Short Physical Performance Battery
TMST: 2-minute step test
WHO: World Health Organization

## Data Availability

Participants information and data sets generated during and/or analyzed during this study will be stored securely and identified by a coded ID number only to maintain participants’ confidentially. Data will be available from the corresponding author on reasonable request.
